# Parental perceptions and predictors of consent for school-located influenza vaccination in urban elementary school children in the United States

**DOI:** 10.1111/irv.12332

**Published:** 2015-08-04

**Authors:** Susan Cheung, Hai-Lin Wang, Laurene Mascola, Alvin Nelson El Amin, Pia S Pannaraj

**Affiliations:** a Division of Infectious Diseases, Children’s Hospital Los AngelesLos Angeles, CA, USA; b Acute Communicable Disease Control, Los Angeles County Department of Public HealthLos Angeles, CA, USA; c Immunization Program, Los Angeles County Department of Public HealthLos Angeles, CA, USA; d Department of Pediatrics and Molecular Microbiology and Immunology, Keck School of Medicine, University of Southern CaliforniaLos Angeles, CA, USA

**Keywords:** Health belief model, influenza, influenza vaccine, school-located influenza vaccination

## Abstract

**Background:**

School-located influenza vaccination (SLV) programs have the potential to mass-vaccinate all enrolled children, but parental consent is required.

**Objective:**

To examine parental attitudes and determine predictors of parental consent for vaccination of schoolchildren through SLV programs.

**Patients/Methods:**

Surveys were distributed to parents of 4517 children during 2009–2010 (year 1) and 4414 children during 2010–2011 (year 2) in eight elementary schools in conjunction with a SLV program.

**Results:**

Participants included 1259 (27·9%) parents in year 1 and 1496 (33·9%) in year 2. Parental consent for 2009 H1N1, 2009 seasonal, and 2010 seasonal influenza vaccines was obtained from 738 (70·8%), 673 (64·5%), and 1151 (77·2%) respondents, respectively. During the 2009 pandemic, respondents concerned about influenza severity were twice as likely to consent for the 2009 H1N1 vaccination compared to unconcerned respondents (OR 2·04, 95% CI:1·19–3·51). During year 2, factors that predicted parental consent were the perception of high susceptibility to influenza infection (OR 2·19, 95% CI:1·50–3·19) and high benefit of vaccine (OR 2·23, 95% CI:1·47–3·40). In both years, college-educated parents were more likely to perceive vaccine risks (year 1: 83·6 versus 61·5%, *P* < 0·001 and year 2: 81·1% versus 60·6%, *P* < 0·001) and less likely to consent for seasonal influenza vaccine (year 1: OR 0·69, 95% CI:0·53–0·89 and year 2: OR 0·61, 95% CI:0·47–0·78) compared to non-college-educated parents.

**Conclusions:**

Parents who appreciate the risks of influenza and benefits of vaccination are more likely to consent for SLV. More research is needed to determine how to address heightened safety concerns among college-educated parents.

## Introduction

Schoolchildren, aged 5–18 years, represent the primary vector of influenza transmission in the community.^1^,^2^ Influenza attack rates of 30–50% among schoolchildren are higher than those of adults, and children have longer periods of communicability compared to adults.^3^ Societal burdens of influenza include excess medical visits, excess antibiotic use, school absenteeism, parental work absenteeism, secondary illness among family members, and mortality.^2^,^4^–^6^ Improving vaccination rates among school-aged children will benefit the vaccinated children and reduce community-wide transmission of influenza.^7^–^10^,^2^ Disease modeling of influenza pandemics suggests that vaccinating schoolchildren may be the most efficient approach to reduce overall numbers of infection.^11^,^12^ For these reasons, the Center for Disease Control and Prevention (CDC)’s Advisory Committee on Immunization Practices expanded the recommendation for annual influenza vaccination to school-age children beginning in the 2008–2009 influenza season.^13^ Since then, annual influenza vaccination coverage has increased yearly but still only reached 55% among children 5–17 years of age during the 2013–2014 season.^14^

School-located mass vaccination (SLV) programs have effectively increased influenza vaccination rates among children.^15^–^17^ These programs increase access by offering vaccines during school hours and do not require parental presence during vaccine administration. However, parents play an important role in influenza prevention as they must consent for their children’s vaccination. Most SLV programs in the United States vaccinate between 15% and 50% of students.^16^,^18^ The SLV programs in Hawaii have achieved high statewide success. However, despite widespread availability, promotion and favorable media attention toward SLV programs, <50% vaccination rate has been achieved.^15^

Obtaining parental consent is a major obstacle for influenza vaccination in public schools.^19^,^20^ Because parental perception influences their decision to allow their children to be vaccinated, it is important to understand these factors in an effort to increase consent rates. We conducted a cross-sectional survey to ascertain parental perceptions of influenza illness and influenza vaccinations and to determine predictors of consent for school-located vaccination in urban Los Angeles County schools over two influenza seasons.

## Methods

### Elementary schools

Surveys were distributed to parents as part of a public health department supported SLV program in eight elementary schools in two Los Angeles County school districts.^17^ The elementary schools enrolled children from pre-kindergarten through 6th grade, ages 5–13 years. Surveys were sent home with each student in the parent’s preferred language of English, Spanish, Chinese, or Vietnamese in September of the 2009–2010 (year 1) and 2010–2011 (year 2) school years. During the pandemic situation in 2009, seasonal and 2009 H1N1 influenza vaccines were offered at all schools through at least one SLV clinic. Influenza prevention information, vaccine information statement, and consent form accompanied the survey. During the year 2, active SLV programs continued at four of the eight elementary schools. The survey was sent home with influenza prevention fliers in schools without SLV programs. Classroom teachers were responsible for collecting the returned forms. If not returned, surveys were sent home again up to three times between September and November each year. One personal phone call and three automated calls were used to remind parents to return the influenza vaccination consent forms and surveys.

### Survey design

Survey questions were developed using constructs from the Health Belief Model,^21^ which suggests that a parent’s decision to have his or her child vaccinated against influenza is based on perceptions regarding the child’s susceptibility to influenza, the severity of disease, and the risks and benefits of influenza vaccination.^22^ Survey participants’ attitudes were assessed using a 3-point scale with an additional “I don’t know” option. Preference for injection with trivalent inactivated influenza vaccine (IIV) or nasal spray with live attenuated influenza vaccine (LAIV) was assessed. The survey also asked parents whether their child received influenza vaccine during the previous year, where their child was vaccinated, and whether any family members were vaccinated. In addition, sociodemographic characteristics of survey participants were assessed. The Institutional Review Boards at Children’s Hospital Los Angeles and Los Angeles County Department of Public Health reviewed and approved this study.

### Statistical analysis

Consent status was determined by return of a signed vaccination consent form in SLV schools or answer to the survey question, “If flu vaccine is offered at your child’s school, would you consent for your child to receive vaccine at school?” in the four schools without SLV programs during year 2. Intention to consent was treated equally to actual parental consent because the parents at those four schools had had experience with the SLV program during the prior school year. Statistical analyses were performed using spss Statistics version 19 (IBM Corp., Armonk, NY, USA) with a two-sided type 1 error of 0·05. Chi-square tests were used to analyze the differences in dichotomized responses and sociodemographic characteristics. Multivariate logistic regression models were used to determine the predictors of respondents’ consent for vaccination.

## Results

### Sociodemographic characteristics

The survey was distributed to 4517 children in year 1 and 4414 children in year 2. Demographic characteristics of enrolled students and survey respondents are shown in Table1. Students of Latino/Hispanic and Asian ethnic origins made up the majority. Among the enrolled students, 79·8% were socioeconomically disadvantaged as defined by the California Department of Education as a student neither of whose parents have received a high school diploma or a student who is eligible for the free or reduced-price lunch program.

**Table 1:** Sociodemographic characteristics of enrolled students and survey respondents by year

Characteristics	2009–2010 *n* (%)		2010–2011 *n* (%)	
	Enrollment	Respondents	Enrollment	Respondents
*Total*	4517	1259 (27·9)	4414	1496 (33·9)
District				
1	2271	901 (39·7)	2250	815 (36·2)
2	2246	358 (15·9)	2164	681 (31·5)
SLV program				
Present	4517	1259 (27·9)	2334	1034 (44·3)
Absent	–	–	2080	462 (22·2)
Race				
Hispanic Latino	3201 (70·9)	586 (48·4)	3129 (70·9)	870 (60·2)
Asian	1191 (26·4)	550 (45·4)	1156 (26·2)	487 (33·7)
White	47 (1·0)	17 (1·4)	52 (1·2)	22 (1·5)
Black/African American	12 (0·3)	10 (0·8)	16 (0·4)	5 (0·3)
American Indian/Alaskan Native	5 (0·1)	4 (0·3)	6 (0·1)	4 (0·3)
Native Hawaiian/Pacific Islander	24 (0·5)	5 (0·4)	22 (0·5)	3 (0·2)
Other	36 (0·8)	39 (3·3)	32 (0·7)	54 (3·8)
Did not answer		48 (3·8)		51 (3·4)
Education level of parent				
Did not complete high school		254 (20·2)		250 (16·7)
Completed high school	Unknown	382 (30·3)	Unknown	462 (30·9)
Attended some college		284 (22·6)		373 (24·9)
Graduated college		268 (21·3)		279 (18·6)
Did not answer		71 (5·6)		132 (8·8)
Grade level of child				
Pre-K through 2nd	2000 (44·2)	511 (40·6)	1973 (44·7)	380 (45·7)
3rd through 6th	2517 (55·8)	669 (55·4)	2441 (55·3)	160 (54·3)
Did not answer		51 (4·1)		5 (0·3)

During year 1, 1445 parents returned their surveys. One hundred and eighty-six surveys from parents of a sibling not enrolled in one of the eight study schools were excluded, leaving 1259 (27·9%) surveys from parents of enrolled students for analysis. During year 2, 1506 parents returned their surveys. Ten surveys from parents of children not enrolled in a study school were excluded leaving 1496 (33·9%) surveys for analysis. The response rate improved in year 2 compared to year 1 (33·7% versus 27·3%; *P* < 0·0001). Response rates were higher in schools with SLV programs compared to those without vaccination programs (44·3% versus 22·2%, *P* < 0·001).

## 2009 H1N1 influenza

**Figure 1:**
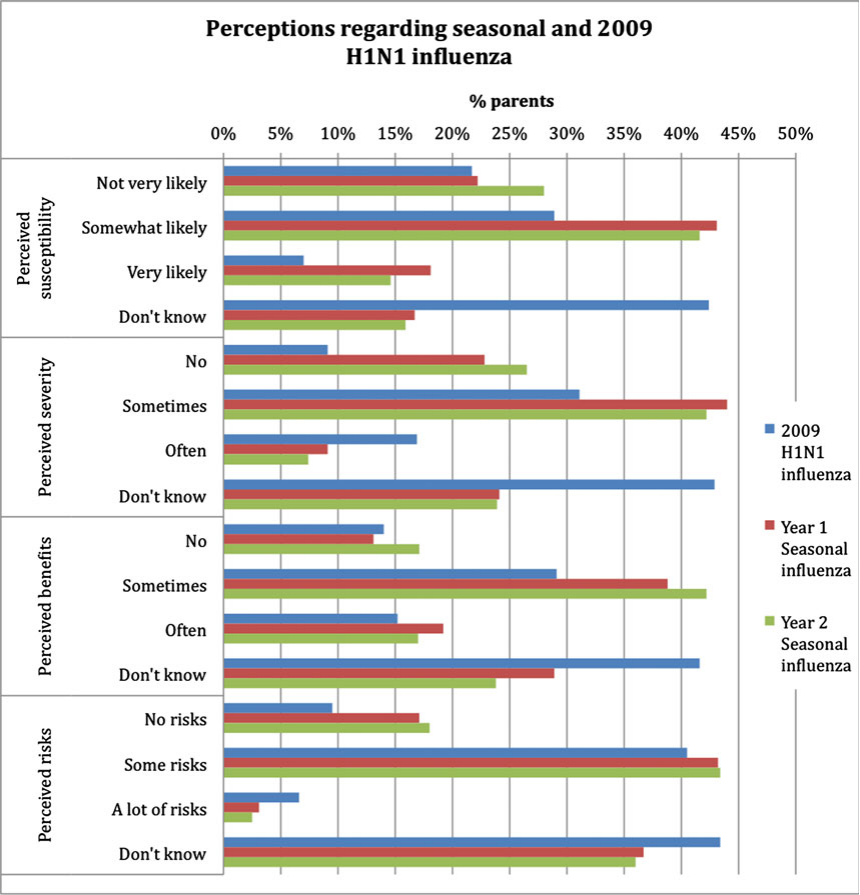
Perceptions of survey respondents regarding the seasonal and 2009 H1N1 influenza illness and vaccine for the 2009–2010 and 2010–2011 seasons.

Perceptions of 2009 H1N1 influenza were assessed separately from seasonal influenza during year 1 because the survey was distributed during the 2009 H1N1 influenza pandemic. Figure1 displays answers to questions regarding perceived susceptibility and severity to influenza illness and perceived benefit and risk of the vaccines. A high number of respondents felt uncertain about 2009 H1N1 influenza illness and vaccination as reflected by answers of “I don’t know” by 42·0–43·8% responders. Perceptions on susceptibility and severity were similar among parents of all races/ethnicities. However, Asian respondents were more likely to believe that vaccination would prevent infection (84·5 versus 67·1%, *P* < 0·001), while Hispanic respondents were more concerned about vaccine safety (85·9 versus 79·0%, *P* = 0·021). College-educated compared to non-college-educated respondents were more likely to believe that the 2009 H1N1 influenza could cause a severe infection (87·2 versus 80·8%, *P* < 0·023), but also were more concerned regarding vaccine safety (91·3 versus 73·7%, *P* < 0·001).

Overall, 948 (75·4%) respondents consented for influenza vaccine during year 1, accounting for 21·0% of enrolled children. Most (60·3%) consented for both 2009 H1N1 and seasonal influenza vaccination; however, 23·5% consented for only 2009 H1N1 and 16·1% consented for only seasonal vaccine. Table2 displays predictors of SLV consent. After adjusting for race in a multivariate regression model, the perception of influenza illness severity remained a significant predictor of vaccine consent.

**Table 2:** Effects of perceptions and sociodemographic characteristics on influenza vaccination consent status in the 2009–2010 and 2010–2011 seasons

Characteristic	2009–2010								2010–2011			
	2009 H1N1 influenza				Seasonal vaccine				Seasonal vaccine			
	Univariate analysis		Multivariate analysis		Univariate analysis		Multivariate analysis		Univariate analysis		Multivariate analysis	
	OR (95% CI)	*P*-value	aOR (95% CI)	*P*-value	OR (95% CI)	*P*-value	aOR (95% CI)	*P*-value	OR (95% CI)	*P*-value	aOR (95% CI)	*P*-value
High perceived susceptibility	1·12 (0·77–1·62)	0·564	–	–	0·99 (0·72–1·36)	0·951	–	–	2·54 (1·93–3·34)	<0·001	2·19 (1·50–3·19)	<0·001
High perceived severity	1·53 (0·95–2·46)	0·082	2·04 (1·19–3·51)	0·010	1·08 (0·78–1·48)	0·653	–	–	0·99 (0·74–1·33)	0·952	–	–
High perceived benefits	2·26 (1·53–3·34)	<0·001	1·38 (0·78–2·42)	0·266	1·12 (0·76–1·66)	0·554	–	–	2·01 (1·48–2·72)	<0·001	2·23 (1·47–3·40)	<0·001
Low perceived risk	0·99 (0·63–1·54)	0·965	–	–	1·35 (0·92–1·98)	0·119	–	–	1·84 (1·29–2·62)	0·001	0·84 (0·54–1·32)	0·453
Hispanic	0·80 (0·60–1·04)	0·104	–	–	0·97 (0·75–1·25)	0·803	–	–	0·66 (0·52–0·85)	0·001	0·84 (0·52–1·30)	0·408
Asian	1·40 (1·07–1·84)	0·015	1·41 (0·91–2·17)	0·124	1·02 (0·79–1·32)	0·877	–	–	1·41 (1·09–1·82)	0·008	1·48 (0·91–2·41)	0·115
Any college education	0·85 (0·65–1·12)	0·240	–	–	0·69 (0·53–0·89)	0·005	0·75 (0·57–0·99)	0·043	0·61 (0·47–0·78)	<0·001	0·55 (0·37–0·81)	0·002
District 1	0·88 (0·65–1·21)	0·429	–	–	1·10 (0·81–1·50)	0·524	–	–	1·85 (1·43–2·38)	<0·001	2·42 (1·66–3·53)	<0·001
SLV Program	–	–	–	–	–	–	–	–	1·18 (0·91–1·53)	0·215	–	–
Grade K-2	1·02 (0·78–1·35)	0·874	–	–	1·14 (0·88–1·48)	0·325	–	–	1·05 (0·83–1·34)	0·680	–	–
Vaccinated Previous Year	0·95 (0·72–1·26)	0·715	–	–	1·59 (1·22–2·08)	0·001	1·56 (1·18–2·06)	0·002	1·68 (1·29–2·18)	<0·001	1·54 (1·04–2·27)	0·030
Family vaccinated	1·032 (0·8–1·36)	0·820	–	–	0·73 (0·56–0·95)	0·017	0·95 (0·68–1·33)	0·749	0·90 (0·69–1·18)	0·449	–	–

### 2009–2010 seasonal influenza

Although perceptions regarding the year 1 seasonal influenza were similar to those of 2009 H1N1 influenza, many more respondents expressed more definite answers (i.e., less “I don’t know” answers) regarding seasonal influenza (Figure1). Perceived influenza illness susceptibility was similar among all races/ethnicities. However, Hispanic respondents were less likely to believe that influenza can cause a severe infection (59·7 versus 78·0%, *P* < 0·001) or that the seasonal influenza vaccine is protective (74·5 versus 87·9%, *P* < 0·001) compared to non-Hispanic respondents. Participants’ education level did not affect perceived influenza susceptibility or severity, but parents who attended college were more likely to perceive risks associated with seasonal influenza vaccination than non-college-educated respondents (83·6 versus 61·5%, *P* < 0·001).

There were 673 (64·5%) survey participants that consented for their child to receive the seasonal influenza vaccine at school, accounting for only 14·9% of student enrollment of all schools. Children of 42 respondents were already vaccinated; 84% of these respondents had attended at least some college. Predictors of parental SLV consent are shown in Table2. In the multivariate analysis, lack of parental college education and the child’s influenza vaccination history remained significant predictors of consent for influenza vaccine to be administered at school. Injection and nasal spray vaccines were equally preferred (IIV 48·3%, LAIV 49·8%, either vaccine 1·9%).

Survey respondents who did not consent for their unvaccinated child to receive vaccine at school during year 1 were asked to free text their reason. Of 56 respondents who provided a reason, 20 (35·7%) preferred to vaccinate at their pediatrician’s office, 16 (28·6%) were concerned for adverse effects, 7 (12·5%) were concerned about their child’s underlying medical condition, 5 (8·9%) believed vaccination was unnecessary, 3 (5·3%) wanted a parent present during vaccination, and 4 (7·1%) expressed other reasons. Respondents who attended at least some college accounted for 68·4% of those who preferred to vaccinate at their pediatrician’s office, but this demographic group also accounted for 56·3% of respondents concerned about adverse effects and 80% of those who believed vaccination was unnecessary.

### 2010–2011 seasonal influenza

Respondents’ perceptions regarding influenza assessed during year 2 are shown in Figure1. Perceptions differed among respondents of different racial/ethnic backgrounds and education levels. Hispanic respondents were less likely than non-Hispanics to believe that influenza could cause a severe infection (58·9% versus 75·1%, *P* < 0·001) or that the influenza vaccine is protective (73·5% versus 84·8%, *P* < 0·001). However, Hispanic respondents were also less likely to perceive risks of vaccination (68·8 versus 76·7%, *P* = 0·010). Respondents who attended college were slightly less likely to perceive vaccination benefit (75·5 versus 80·8%, *P* = 0·038) and significantly more likely to perceive vaccination risks (81·1 versus 60·6%, *P* < 0·001) compared to those who did not attend any college.

During year 2, 1151 (77·2%) survey respondents consented or would have consented if SLV was available to have their child receive influenza vaccine at school, representing 26·1% of student enrollment. Children of 132 respondents were already vaccinated; education level of these respondents was not significantly different. However, perceptions and college education did influence SLV consent status (Table2). In the multivariate analysis, high perceived disease susceptibility, high perceived vaccination benefit, lack of college education, and the child’s previous influenza vaccination remained significant predictors of vaccination consent. Most respondents preferred the intranasal vaccine to the injection vaccine (63·1 versus 36·9%, *P* < 0·001).

Respondents who did not consent for their unvaccinated child to receive vaccine at school were asked to free text their reason. Of 140 respondents who provided a reason, 68 (48·6%) preferred to vaccinate at their pediatrician’s office, 25 (17·9%) were concerned for adverse effects, 26 (18·6%) believed vaccination was not necessary, 7 (5·0%) were concerned about their child’s underlying medical condition, 6 (4·3%), wanted a parent present during vaccination, and 8 (5·7%) expressed other reasons. Respondents who attended at least some college accounted for 54·8% of those who preferred to vaccinate at their pediatrician’s office, but this demographic group also accounted for 60·9% of respondents concerned about adverse effects and 68·0% of those who believed vaccination was unnecessary.

### Changes between year 1 and year 2

Statistically significant decreases were seen from year 1 to year 2 in perceived seasonal influenza susceptibility (73·4 versus 66·8%, *P* = 0·001) and severity (70·0 versus 65·2, *P* = 0·019) and perceived vaccination benefits (81·6 versus 77·6%, *P* = 0·028). However, a larger proportion of school enrollment consented or would have consented if SLV was available for influenza vaccine in year 2 than for either influenza vaccine in year 1 (26·1 versus 21·0%, *P* < 0·001). More family members also received influenza vaccine in year 2 (69·1 versus 52·1%, *P* < 0·001); reported vaccination in mothers, fathers, and siblings increased, whereas grandparent vaccinations remained steady. Preference for the intranasal vaccine for children increased in year 2 (63·1 versus 48·3%, *P* < 0·001).

## Discussion

This survey of parents, conducted in a large urban community, found that perceptions of influenza illness susceptibility, influenza severity, and potential vaccine benefit are significant predictors of parental consent for school-located influenza vaccination of elementary school children. Vaccine safety is an important concern, specifically among college-educated respondents. Despite these perceptions, vaccine consent did increase significantly over the two school years surveyed.

During the 2009–2010 school year, parents expressed a significant amount of uncertainty toward influenza, especially with respect to the 2009 H1N1 influenza vaccine. This finding reflected public perception during the influenza pandemic.^23^,^24^ A large contributor to the uncertainty may have been due to the contradictory messages delivered by the US media, with some questioning the effectiveness of preventive measures while others overemphasized deaths related to influenza.^25^ Nevertheless, fear of the potential severity of the 2009 H1N1 influenza was an important factor in parental consent for the 2009 H1N1 vaccine. Fortunately, the 2009 pandemic was not as severe as initially feared.^26^ During the 2010–2011 season, illness severity was no longer a primary concern. Rather, survey respondents who perceived a high likelihood of acquiring influenza or believed the vaccine would protect against disease were twice as likely to consent for SLV.

A key demographic factor that proved to be a significant predictor of seasonal influenza vaccine consent for vaccination at school was the respondent’s education level. Respondents who attended any college were one and a half times less likely to give parental consent to vaccinate their children through the SLV program. Although college-educated respondents were more likely to prefer vaccination at their healthcare provider’s office during year 1, education level did not appear to influence plans to vaccinate outside of the SLV program during year 2. In both years of the study, college-educated respondents were significantly more likely to perceive risks associated with vaccination compared to non-college-educated respondents. Vaccine safety concerns have been implicated as an important reason by parents who choose not to vaccinate their child in multiple studies of infants, school-aged children, and adolescents.^22^,^27^–^31^ Our study showed that the safety concern is heightened among college-educated respondents. Parental vaccine refusal is increasing nationwide, especially among parents with college degrees and higher socioeconomic status.^32^,^33^ Further investigation is needed to understand this vaccination hesitancy in highly educated parents and how to overcome concerns about the vaccine safety. Educated parents may have more access and time to investigate websites and media sources that discuss vaccine safety issues, including those sites with incorrect information. Interventions targeted at this group should include more information about how to interpret media and online vaccine discussions with appropriate website referral. It is also possible that educated parents may fear communication challenges or loss of parental control if their children are vaccinated at school.^34^ Healthcare providers’ offices are perceived to be better prepared over SLV in case of side effects.^31^

Rates of parental consent or intention to consent for their child’s vaccination increased in the second year of our study despite decreases in the perceptions of influenza disease susceptibility, disease severity, and benefit of vaccine from the pandemic year. Having a free-of-charge influenza vaccination program located on site at school provided easy access and eliminated common barriers of competing time demands, cost, and inconvenience cited in other studies.^22^,^28^ Because SLV clinics were offered at all eight study schools during year 1, it is possible that influenza vaccination became increasingly viewed as a social norm. Many investigators have shown that peer influence is an important determinant of vaccine uptake.^22^,^35^,^36^ SLV programs serve as an excellent setting to promote influenza vaccination as a social norm. The presence of our SLV program likely had a role in reminding and encouraging parents to have their children vaccinated.

Preference for the intranasal vaccine also increased in the second year such that nearly two-thirds opted for the intranasal spray if their child could receive either form of vaccination. Several large pediatric studies suggest that LAIV is more effective than IIV.^37^ However, more comparisons are obligatory as circulating strains change year to year.^38^ Local mucosal delivery and induction of diverse T-cell responses by LAIV in children is believed to contribute to protection.^39^ In addition, LAIV shows high efficacy when epidemic influenza viruses were not well matched to the vaccine strains in vaccinated schoolchildren and offered herd protection for the community.^40^ Nevertheless, offering both vaccines in SLV programs is important to provide options for children with underlying conditions who cannot receive LAIV.

This study has several limitations. The study was conducted in two urban school districts where the predominant ethnicity was Hispanic followed by Asian. Nearly 80% of the schoolchildren were from socioeconomically disadvantaged families. Although our findings may not be generalizable to all other communities, these results may be applied to other low-income and predominantly Hispanic populations in the United States. The low response rates clearly represent a selection bias. The higher vaccine consent rates among survey participants compared to all school enrollees indicate that parents who strongly supported vaccination for their child were more motivated to return their completed surveys and thereby skewing the responses. Therefore, the results reported here are specifically the analyses of the survey respondents and may not reflect the perceptions of all parents. The influenza prevention information and/or influenza vaccine information statements sent home with the survey may have influenced survey responses. However, the large numbers of “I don’t know” answers suggest that these information sheets were not sufficient. Overcounting of parental responses of siblings who returned more than one survey is possible. Finally, we specifically evaluated SLV consent rates. Our survey did not specifically query for concerns related to receiving a vaccine at the school versus receiving an influenza vaccine at a healthcare provider’s office.

Many variables factor into the decision-making process of parents to consent their schoolchildren for participation in SLV programs. This study found that in the season following the 2009 pandemic, influenza susceptibility and vaccine efficacy were the most important factors associated with parental consent. Our findings suggest that interventions targeted at parents to help increase their understanding of their children’s risk of acquiring influenza and benefit of vaccination may improve SLV program vaccination rates. In addition, tailored interventions about vaccine safety are needed for college-educated parents. Community-centered education programs and text-messaging interventions targeted toward parents have led to increase in influenza vaccination rates,^41^ but others have found that pro-vaccine messages can increase misperceptions or reduce influenza vaccination intention.^42^ More research is needed on the best methods to change parental perceptions in order to truly increase the participation rates among SLV programs in elementary schools.
